# Nutritional Values of Industrial Hemp Byproducts for Dairy Cattle

**DOI:** 10.3390/ani12243488

**Published:** 2022-12-10

**Authors:** Yiqiang Wang, Jianxu Gao, Chuanteng Cheng, Jingyi Lv, Modinat Tolani Lambo, Guangning Zhang, Yang Li, Yonggen Zhang

**Affiliations:** College of Animal Science and Technology, Northeast Agricultural University, Harbin 150030, China

**Keywords:** nutritional value, industrial hemp byproducts, dairy cattle

## Abstract

**Simple Summary:**

At present, the price of conventional feed ingredients in the world fluctuates greatly, and the economic outcomes of many farms are facing this challenge. At the same time, with the relaxation of the planting restriction policy, the industrial hemp planting area has increased, and the processing of industrial hemp byproducts has become a great challenge. In this study, we compared the chemical composition, carbohydrate and protein composition, *in situ* ruminal degradability, intestinal digestibility, and available energy values of industrial hemp byproducts and conventional feeds to make up for the gaps in the data of industrial hemp byproducts and provide theoretical support for the application of industrial hemp byproducts in dairy cattle production.

**Abstract:**

The objective of this experiment was to explore the nutritional components of industrial hemp byproducts (industrial hemp ethanol extraction byproduct, IHEEB; industrial hemp stalk, IHS; industrial hemp seed meal, IHSM; industrial hemp oil filter residue, IHOFR) and provide theoretical support for the application of industrial hemp byproducts in dairy cattle production. This experiment used a combination of a wet chemical method with Cornell Net Carbohydrate and Protein System, *in situ* nylon bag technique, and three-step *in vitro* method to compare the chemical composition, carbohydrate and protein composition, *in situ* ruminal degradability and intestinal digestibility of industrial hemp byproducts and conventional feeds (alfalfa hay, AH; soybean meal, SBM). Available energy values were estimated based on the National Academies of Sciences, Engineering, and Medicine. The results showed that the nutritional composition of different feeds varied greatly. The two types of IHEEB were enriched with ash, crude protein (CP), neutral detergent fiber (NDF), and calcium, while the contents of neutral detergent insoluble crude protein, acid detergent insoluble crude protein, and acid detergent lignin were higher. As a result, the non-degradable carbohydrate and protein components were higher, and the effective degradation rate of rumen dry matter and protein was lower. IHS contains higher non-protein nitrogen and NDF, which enables it to provide more CP rumen effective degradation rate and carbohydrates, but the high acid detergent fiber also limits its application. IHSM possesses 296 g/kg CP and high rumen undegradable protein and intestinal digested protein, which can provide rumen bypass protein in dairy cows, making it a potentially good protein source. IHOFR had higher ether extract, rumen available protein degradation rate, and total tract digested protein, which can provide more energy and easily degradable protein for lactating cows. The available energy value of IHEEB and IHS was lower than AH, while SBM is between IHFOR and IHSM. In addition, the tetrahydrocannabinol of three industrial hemp byproducts that have not been assessed by the European Food Safety Authority (EFSA) was tested to evaluate their safety, and all of them were less than the limit set by ESFA. In conclusion, industrial hemp byproducts can be considered for inclusion in dietary formulations as unconventional feed sources for dairy cattle, but the purpose of use needs to be properly considered.

## 1. Introduction

Currently, the price of conventional feed ingredients in the world fluctuates greatly, and the economic benefits of many farms face this challenge. Annually, there are many byproducts from agricultural processing worldwide, and their rich nutrients and affordable prices gradually make them a focus of attention for nutrition experts [1,2]. Industrial hemp is defined as an annual herb of the cannabis genus in the cannabis family [3], whose flowers, seeds, stems, leaves, and roots contain less than 0.3% tetrahydrocannabinol (THC). At the same time, along with the relaxation of the planting restriction policy, the industrial hemp planting area has increased, and industrial hemp byproduct processing has become a thorny problem [4]. Such agricultural byproducts with high nutritional value can usually be used by ruminants and converted into animal products. Therefore, the application of industrial hemp byproducts in animal feed may be a good solution [5].

Industrial hemp is a multipurpose plant, such as seeds for oil extraction, stalks for fiber extraction, flowers and leaves for drug extraction, and so on [6]. Regardless of the purpose of planting, byproducts such as leaves, stalks, and seed cakes are produced. Previous studies on the byproducts of industrial hemp mainly focus on industrial hemp seed cake, which is believed to be a high-quality protein source for dairy ruminants with a crude protein (CP) content of 344 g/kg [7]. Moreover, it has been reported that industrial hemp seed cake has better rumen bypass protein than rapeseed meal and is a good rumen protection protein source for ruminants [8]. In previous studies, industrial hemp seed cake was fed to ruminants, and the results showed that adding a certain amount to the diet increased milk yield and milk fat content and improved milk quality [9]. In addition, industrial hemp leaf contains 130 g/kg CP, 89 g/kg ether extract (EE), 212 g/kg ash, and 447 g/kg neutral detergent fiber (NDF), which can provide rich nutrients for ruminants [10]. It can be seen that the byproducts of industrial hemp have high nutritional value and different byproducts have different characteristics, so it has a good application prospect as ruminant feed. Few studies have been reported on other conventional byproducts of industrial hemp processing, such as ethanol extraction byproduct, stalk, and oil filter residue, except those mentioned above [10,11]. Currently, there is no systematic study on nutrient composition analysis and digestion and degradation characteristics of common industrial hemp byproducts, which will limit their application as feed in actual livestock production [8,10,11,12].

Cornell Net Carbohydrate and Protein System (CNCPS) can reflect the rumen degradation rate, digestibility, and energy and protein absorption efficiency of feed in dairy cows [13]; it can also scientifically evaluate the nutritional value of carbohydrate and protein in feed [13]. The digestibility of NDF and CP, important indicators to determine the value of feeds, differs from feed to feed. The appropriate content of NDF and CP can stimulate rumination and saliva production, thus buffering the rumen and promoting rumen health [14]. The *in situ* nylon bag technique (ISNBT) and three-step *in vitro* method (TSIVM) are often used to simulate the prediction of nutrient degradability and digestibility in animal production and feed value [15,16]. Alfalfa hay and soybean meal are used as common conventional feed in dairy farms. Comparing them with industrial hemp byproducts can more clearly show the nutritional characteristics of the latter.

Therefore, the objectives of this study were to compensate for the gaps in data on industrial hemp byproducts by using a combination of a wet chemical method with CNCPS, ISNBT and TSIVM to compare the chemical composition, carbohydrate and protein composition, *in situ* ruminal degradability, and intestinal digestibility of industrial hemp byproducts and conventional feeds. In addition, the digestible energy values of all feeds were estimated according to the guidelines of the National Academies of Sciences, Engineering, and Medicine (NASEM). As a result, this study will enrich the database of unconventional feed resources and provide theoretical support for applying industrial hemp byproducts in dairy cattle production.

## 2. Materials and Methods

All animal experiments were approved by the Northeast Agricultural University Animal Science and Technology College Animal Care and Use Committee (Protocol number: NEAUEC20181007).

### 2.1. Sample Collection

Zhaozhou industrial hemp ethanol extraction byproduct (ZIHEEB) was provided by Heilongjiang Zhongsheng Biotechnology Co., Ltd. (Daqing, China). Daxing’ anling industrial hemp ethanol extraction byproduct (DIHEEB), industrial hemp seed meal (IHSM), and industrial hemp oil filter residue (IHOFR) were provided by Daxing’ anling Jinshan Industrial Hemp Biotechnology Co., Ltd. (Daxing’ anling, China). Industrial hemp stalks (IHS) were collected from Heihe City, Heilongjiang. Alfalfa hay (AH) and soybean meal (SBM) were provided by Heilongjiang Wellhope Animal Husbandry Co., Ltd. (Harbin, China).

The industrial hemp ethanol extraction byproduct used in this study was the residue of the flower and leaf parts of hemp after ultrasound-assisted extraction of cannabidiol (CBD) in ethanol; IHS was the stalk from the top of the plant that has no use value in the process of industrial hemp fiber; IHSM was obtained by processing the oil through industrial hemp seeds using a physical cold pressing method; and IHOFR was the filtration residue from hemp seed oil production. The samples (n = 5) were collected from corresponding feeds and used as replicates.

### 2.2. Chemical Analysis

All samples were dried at 55 °C for 48 h, ground through a 1-mm screen, then stored at 4 °C until used for subsequent chemical composition analysis. They were analyzed for the contents of dry matter (DM; method 930.15), CP (method 976.05), EE (method 920.39), and ash (method 942.05) according to AOAC International [17]. The soluble crude protein (SCP), non-protein nitrogen (NPN), neutral detergent insoluble crude protein (NDICP), and acid detergent insoluble crude protein (ADICP) were determined according to the procedure recommended by Licitra et al. [18]. NDF, acid detergent fiber (ADF), and acid detergent lignin (ADL) were analyzed using Ankom 220 Fiber Analyzer (Ankom Technology Corp., Macedon, NY, USA), according to the methods of Van Soest [19]. Starch content was measured using the Megazyme Total Starch Assay Kit (K-TSTA; Megazyme International Ireland Ltd., Bray, Ireland). Non-fibrous carbohydrate (NFC) content was calculated using the following formula:

NFC (g/kg DM) = 1000 − NDF (g/kg DM) − CP (g/kg DM) − EE (g/kg DM) − Ash (g/kg DM).(1)

The concentration of calcium (Ca) was analyzed using permanganate titration described by the National Standard of the People’s Republic of China: Determination of Calcium in Feed (GB/T 6436-2002), and the concentration of phosphorus (P) was analyzed using spectrophotometry described by Determination of Phosphorus in Feeds-Spectrophotometry (GB/T 6437-2002) [16]. THC of the three industrial hemp byproducts (ZIHEEB, DIHEEB, and IHS) that have not been assessed by the European Food Safety Authority (EFSA) were analyzed by a high-performance liquid chromatograph with an ultraviolet detector (LC-20A; Shimadzu Corp., Kyoto, Japan) [20]. The lower limit of detection (LOD) and lower limit of quantification (LOQ) of cannabinoids was 0.03 mg/kg and 0.1 mg/kg.

### 2.3. Protein and Carbohydrate Fractions

CNCPS divides feed protein into A, B1, B2, B3, and C fractions. The fractions, in turn, represent non-protein nitrogen (PA), rapidly degraded protein (PB1), intermediately degraded protein (PB2), slowly degraded protein (PB3), and unavailable protein (PC). Carbohydrate fractions are described using a scheme similar to that used for proteins. Carbohydrates (CHO) are further categorized into A, B1, B2, and C fractions. The fractions, in turn, are sugars and soluble fraction (CA), starch and pectin (CB1), fraction available cell wall (CB2), and unavailable cell wall (CC). The formula for calculating the relevant components is as follows [21]:

PA (g/kg CP) = NPN (g/kg SCP) × 0.001 × SCP (g/kg CP),(2)

PB1 (g/kg CP) = SCP (g/kg CP) − PA (g/kg CP),(3)

PB2 (g/kg CP) = 1000 − PA (g/kg CP) − PB1 (g/kg CP) − PB3 (g/kg CP) − PC (g/kg CP),(4)

PB3 (g/kg CP) = NDIP (g/kg CP) − ADIP (g/kg CP),(5)

PC (g/kg CP) = ADIP (g/kg CP),(6)

CHO (g/kg DM) = 1000 − CP (g/kg DM) − EE (g/kg DM) − Ash (g/kg DM),(7)

CC (g/kg CHO) = 1000 × [NDF (g/kg DM) × 0.001 × ADL (g/kg NDF) × 2.4]/CHO (g/kg DM),(8)

CB2 (g/kg CHO) = 1000 × [(NDF (g/kg DM) − NDIP (g/kg CP) × 0.001 × CP (g/kg DM) − NDF (g/kg DM) × 0.001 × ADL (g/kg NDF) × 2.4)]/CHO (g/kg DM),(9)

NSC (g/kg CHO) = 1000 − CB2 (g/kg CHO) − CC (g/kg CHO),(10)

CB1 (g/kg CHO) = Starch (g/kg NSC) × [1000 − CB2 (g/kg CHO) − CC (g/kg CHO)]/1000,(11)

CA (g/kg CHO) = [1000 − Starch (g/kg NSC)] × [l000 − CB2 (g/kg CHO) − CC (g/kg CHO)]/1000.(12)

### 2.4. In Situ Rumen Degradation

*In situ* rumen degradation of DM, NDF, and CP of all samples was determined in 3 Holstein cows (BW = 613 ± 10.2 kg, mean ± SD) with a rumen fistula as described by Li et al. [22]. The basal diet (% of DM) consisted of 41.5% concentrate mixture, 15.8% corn silage, and 42.7% Chinese wildrye hay and was fed twice daily with a total DMI of 15 g/kg of BW. The samples were dried at 55 °C and then ground to pass through a 2-mm screen by a Wiley mill (Arthur H. Thomas Co., Swedesboro, NJ, USA). Then, 7 g of the sample was weighed, put into nylon bags (10 × 20 cm, 40-µm pore size; Ankom Technology, Macedon, NY, USA), and tightened with a rubber band. Before *in situ* rumen degradation, all nylon bags containing samples were placed in a larger mesh bag (36 × 42 cm); this ensured that all nylon bags were placed below the particulate mat layer in the ventral sac of the rumen. Samples were made in two parallel cultures in the rumen of each cow for 0, 2, 4, 8, 12, 16, 24, 36, and 48 h. ZIHEEB, DIHEEB, IHS, and AH were incubated *in situ* for an additional 72 h. Following rumen incubation, all nylon bags were cleaned with cold water and then placed in an oven at 55 °C for 48 h before weighing. The residues were transferred into a zip lock bag after sieving with a 1-mm mesh [23]. Finally, the feed residue samples were analyzed for DM, NDF, and CP.

The feeds’ *in situ* degradation constants of DM, CP, and NDF were estimated and described by Ørskov and McDonald [24]; the formula is as follows:

*p* = *a* + *b* (1 − e^−*ct*^) (13)

*p*, the rate of disappearance at time *t* (h); *a* the rapidly degradable fraction; *b*, the potentially degradable fraction; *c*, the rate of *b*.

The effective degradability (*ED*) was calculated as:

*ED* = *a* + *bc*/(*c* + kp) (14)

*a*, *b,* and *c* are constants described above. Assuming a pass rate (kp) of 45 g/kg h^−1^ (ZIHEEB, DIHEEB, IHS, and AH) or 60 g/kg h^−1^ (IHSM, IHOFR, and SBM) [25].

### 2.5. Intestinal Digestion

The intestinal digestibility of the collected residues incubated *in situ* in the rumen for 16 h was determined by the three-step *in vitro* digestion method [26]. First, the residues were passed through a 1-mm screen Wiley mill. Then, 1 g of residues was weighed and placed in a nylon bag. Nylon bags were placed in HCl solution (pH = 1.9) containing 1 g pepsin (sigma, P-7000, Sigma Chemical, St. Louis, MO, USA) and incubated at 39 °C for 1 h. After incubation, the bags were rinsed under cold water until the water was clean. Finally, bags were placed in 1 L solution (pH = 7.75, KH_2_PO_4_ buffer) containing 50 µg thymol and 3 g trypsin (sigma P-7545, sigma Chemical, St. Louis, MO, USA) and incubated in a shaking incubator at 39 °C for 24 h. Afterward, the sample bags were rinsed again and dried at 55 °C for 48 h before the N content was analyzed.

### 2.6. Energy Value Estimation

The fatty acid (FA), residual organic matter (ROM), gross energy (GE), digested proportion of NDF (dNDF), the digested proportion of CP (dCP), digestible energy (DE), total digestible nutrient (TDN), metabolic energy (ME), net energy for maintenance (NE_m_), and net energy for gain (NE_g_) values of all feeds were calculated using a summary approach according to NASEM (2021 and 2016) [27,28]. The feed’s gross energy value was also determined using a bomb calorimeter (Parr 6400; Parr Instruments Co., Moline, IL, USA).

### 2.7. Statistical Analyses

According to the characteristics of byproducts, they were divided into two groups to compare with the conventional feeds: (1) ZIHEEB, DIHEEB, IHS, and AH, (2) IHSM, IHOFR, and SBM. All data in this experiment were analyzed by SAS software (version 9.4, SAS Institute Inc., Cary, NC, USA). The ruminal *in situ* degradation kinetics (*a*, *b*, *c*) were estimated using the NLIN procedure. The data of chemical composition, crude protein and carbohydrate fractions, *in situ* ruminal degradation kinetics, intestinal degradation ratio, and energy values were subjected to a one-way ANOVA procedure, according to the model Y_ij_ = µ + T_i_ + e_j_, where Y_ij_ was the dependent variable, µ was the overall mean, T_i_ was the treatment effect, and e_j_ was the error term. The differences between least square means were assessed using Tukey’s test. The significant differences were stated at *p* < 0.05.

## 3. Results

### 3.1. Chemical Composition

The chemical composition of industrial hemp byproducts and conventional feeds is listed in Table 1. By comparison, it was found that there were great differences in the nutritional composition of various feeds. The ash contents of industrial hemp ethanol extraction byproducts were higher than that of alfalfa hay (*p* < 0.05). Industrial hemp oil filter residue contained the highest EE of 216 g/kg DM, and the CP contents of industrial hemp byproducts ranged from 91.2 g/kg DM (industrial hemp stalk) to 442 g/kg DM (industrial hemp oil filter residue). All industrial hemp ethanol extraction byproducts had lower SCP content than alfalfa hay (*p* < 0.05). The NPN and ADL contents of industrial hemp seed meal and industrial hemp oil filter residue were higher than those of soybean meal (*p* < 0.05). The NDF and ADF contents of industrial hemp ethanol extraction byproducts were similar and lower than those of alfalfa hay (*p* < 0.05). Industrial hemp ethanol extraction byproducts and industrial hemp stalk had higher ADL content than alfalfa hay (*p* < 0.05). The starch content of Daxing’ anling industrial hemp ethanol extraction byproduct was higher than industrial hemp stalk and alfalfa hay (*p* < 0.05), while the Ca and P contents of industrial hemp ethanol extraction byproducts were significantly higher than alfalfa hay (*p* < 0.05). The THC content of industrial hemp ethanol extraction byproducts and industrial hemp stalk was less than the LOQ.

### 3.2. Protein and Carbohydrate Fractions

As shown in Table 2, industrial hemp seed meal had a higher PA value than soybean meal (*p* < 0.05) but significantly lower than that of industrial hemp oil filter residue (*p* < 0.05), while the PA content of the other byproducts was less than that of alfalfa hay (*p* < 0.05). The PB1 content of soybean meal was higher than that of industrial hemp oil filter residue and industrial hemp seed meal (*p* < 0.05). The PB2 was similar between industrial hemp oil filter residue and soybean meal, except for the higher values of industrial hemp seed meal compared to soybean meal (*p* < 0.05). The PB2 content of Zhaozhou industrial hemp ethanol extraction byproduct and industrial hemp stalk was similar to that of alfalfa hay and significantly higher than that of Daxing’ anling industrial hemp ethanol extraction byproduct (*p* < 0.05). Industrial hemp ethanol extraction byproducts had the highest PB3 content, which was significantly higher than alfalfa hay (*p* < 0.05). Industrial hemp byproducts had higher PC values (*p* < 0.05) than alfalfa hay and soybean meal, and only the CHO content of industrial hemp stalk was higher than that of alfalfa hay (*p* < 0.05). Among the carbohydrate fractions, industrial hemp oil filter residue and soybean meal had a higher CA content than industrial hemp seed meal (*p* < 0.05), and there was no significant difference in CA between Zhaozhou industrial hemp ethanol extraction byproduct and alfalfa hay (*p* > 0.05), which was significantly lower than Daxing’ anling industrial hemp ethanol extraction byproduct (*p* < 0.05). In the CB1 component, Daxing’ anling industrial hemp ethanol extraction byproduct content was the highest (*p* < 0.05), and industrial hemp seed meal and industrial hemp oil filter residue were significantly lower than soybean meal (*p* < 0.05). The CB2 was similar between industrial hemp stalk and alfalfa hay, except for the lower values of industrial hemp ethanol extraction byproducts compared to them (*p* < 0.05). The CC contents of industrial hemp ethanol extraction byproducts were significantly higher than that of alfalfa hay (*p* < 0.05).

### 3.3. In Situ Ruminal Degradation

The *in situ* ruminal degradation kinetics in all feeds are shown in Table 3. First, regarding the *in situ* DM rumen degradation kinetics, the *a* value of industrial hemp oil filter residue was 559 g/kg, much larger than industrial hemp seed meal and soybean meal (*p* < 0.05). Daxing’ anling industrial hemp ethanol extraction byproduct had significantly higher *b* and *a* + *b* values than alfalfa hay (*p* < 0.05), and *c* values of industrial hemp ethanol extraction byproducts and industrial hemp stalk were significantly lower than that of alfalfa hay (*p* < 0.05). Industrial hemp oil filter residue contained the highest values of *a* and *ED* than industrial hemp seed meal and soybean meal in *in situ* CP rumen degradation kinetics (*p* < 0.05). Industrial hemp ethanol extraction byproducts and industrial hemp stalk had lower *ED* values of CP than alfalfa hay (*p* < 0.05); as a result, they had higher RUP values (*p* < 0.05). Similarly, industrial hemp seed meal has a higher RUP value than soybean meal (*p* < 0.05). Finally, industrial hemp ethanol extraction byproducts showed significantly higher *a* + *b* values than alfalfa hay (*p* < 0.05) in *in situ* NDF rumen degradation kinetics. Industrial hemp oil filter residue had significantly higher *c* values than soybean meal (*p* < 0.05) and the *ED* value of NDF was rather similar between Zhaozhou industrial hemp ethanol extraction byproduct and alfalfa hay (*p* > 0.05), except for the higher values of Daxing’ anling industrial hemp ethanol extraction byproduct compared to them (*p* < 0.05).

### 3.4. Intestinal Digestion

Table 4 shows the results of the intestinal degradation ratio. The IDDM value of soybean meal was much higher than industrial hemp seed meal and industrial hemp oil filter residue (*p* < 0.05). Similarly, soybean meal has the highest IDRUP value than them (*p* < 0.05). Industrial hemp ethanol extraction byproducts and industrial hemp stalk had lower IDDM and IDRUP values than alfalfa hay (*p* < 0.05). For IDP values, soybean meal and industrial hemp seed meal had non-significant differences (*p* > 0.05), and industrial hemp ethanol extraction byproducts and industrial hemp stalk were smaller than alfalfa hay (*p* < 0.05). Overall, the TTDP value of industrial hemp oil filter residue was the highest, followed by soybean meal and then industrial hemp seed meal (*p* < 0.05). The TTDP values of industrial hemp ethanol extraction byproducts and industrial hemp stalk were smaller than alfalfa hay (*p* < 0.05).

### 3.5. Energy Value Estimation

As shown in Table 5, the GE value of Daxing’ anling industrial hemp ethanol extraction byproduct was lower than that of Zhaozhou industrial hemp ethanol extraction byproduct, industrial hemp stalk, and alfalfa hay (*p* < 0.05), and the GE value of industrial hemp oil filter residue was the highest compared with that of industrial hemp seed meal and soybean meal (*p* < 0.05). Comparison of energy values among the feed ingredients showed the same pattern for all of the energy values (DE, ME, and NE), with Daxing’ anling industrial hemp ethanol extraction byproduct having the lowest energy values than Zhaozhou industrial hemp ethanol extraction byproduct, industrial hemp stalk, and alfalfa hay (*p* < 0.05), whereas industrial hemp oil filter residue had the highest values compared to industrial hemp seed meal and soybean meal (*p* < 0.05), except that the DE value of two kinds of industrial hemp ethanol extraction byproducts was not significantly different for dairy cattle (*p* > 0.05).

## 4. Discussion

### 4.1. Chemical Composition

Routine nutritional evaluation of feed is the basis of the development and utilization of feed resources, and its content reflects the nutritional value of feed. With the gradual opening of the global policy on industrial hemp and the resultant increase in the planting area, more and more industrial hemp processing byproducts are produced [4]. Therefore, this study analyzes the nutritional value of industrial hemp byproducts for the first time, which is of great significance for applying industrial hemp byproducts in animal feed. In this study, the ash and Ca contents of the two kinds of industrial hemp ethanol extraction byproducts were high, consistent with the high Ca content in leaves and flower parts of industrial hemp plants found by Bernstein et al. [29]. We found that two processing byproducts of industrial hemp seed contained high EE, possibly because hemp seed and its product had high levels of n-3 and n-6 fatty acids [30]. Bailoni et al. reported that the fatty acid composition of hemp oil could transfer polyunsaturated fatty acids, especially n-3 fatty acids, to the milk of dairy ruminants [30]. Industrial hemp seed meal has a CP level of 296 g/kg DM, and its nutritional composition is similar to the results of Serrapica et al. [31], making it a potentially good source of protein. Industrial hemp ethanol extraction byproducts and industrial hemp oil filter residue all contain high CP content, making them possible to become potential unconventional feeds. Industrial hemp stalk contains low CP, and high NPN may provide less rapidly degraded protein for animals, and its high NDF can provide abundant carbohydrates for ruminants to maintain normal rumen fermentation, although the high ADF of industrial hemp stalk will limit its application to a certain extent. The higher NDICP of industrial hemp ethanol extraction byproducts and industrial hemp seed meal will make them have more slowly degraded proteins and provide more rumen bypass proteins for ruminants. Compared with the results of Wang et al. [15], the nutrient composition of alfalfa hay in this experiment showed that CP content was low, and NDF and ADF content was high, which may be caused by the late harvest period. Although the NDF of industrial hemp ethanol extraction byproducts in this study was 471 g/kg DM and 460 g/kg DM, respectively, less than alfalfa hay, they could also provide abundant carbohydrates for ruminants. The higher NFC content of Daxing’ anling industrial hemp ethanol extraction byproduct may be related to the higher starch content, which can provide more energy for ruminants [7]. In this experiment, the low CP content of soybean meal may be related to the variety, compared with the study of Yang et al. [32]. In addition, the THC of three industrial hemp byproducts that the EFSA has not assessed was tested to evaluate their safety, and all of them were less than the limit set by ESFA [33]. The above nutrients indicate that the byproducts of industrial hemp have their characteristics and can be regarded as potentially valuable dairy cattle feed.

### 4.2. Protein and Carbohydrate Fractions

CNCPS is a feed analysis mathematical model that links feed value and nutritional requirements of ruminants [34]. Among the protein components, PA is a good nitrogen source for ruminants, and rumen microorganisms can use the ammonia-N rapidly transformed by PA to synthesize high-quality microbial proteins [34]. Compared with alfalfa hay, industrial hemp ethanol extraction byproducts and industrial hemp stalk contain less PA, indicating that they contain more true protein components. PB1 is a true protein that degrades rapidly and almost completely in the rumen [34], while PB2 is a moderately degraded protein and is partially degraded in the rumen [34]. Zhaozhou industrial hemp ethanol extraction byproduct contained more true protein (PB1 + PB2 + PB3) at 704.7 g/kg CP, much higher than alfalfa hay. PC stands for non-degradable nitrogen with ADICP as the main component, which is considered to be the protein part of the feed that ruminants cannot use efficiently [35]. Compared with conventional feed, industrial hemp byproducts had higher PC values except for industrial hemp oil filter residue, especially Daxing’ anling industrial hemp ethanol extraction byproduct, which will reduce its protein degradation rate in ruminants. Compared with soybean meal, industrial hemp oil filter residue contains higher PA and lower PB1, which is more likely to be rapidly degraded by ruminants in the rumen, while industrial hemp seed meal contains lower PB1 and higher PB2 and PC, which is relatively slow to degrade in the rumen, and more proteins could escape the rumen degradation. Although the degradation effect of industrial hemp stalk protein in the rumen is limited, it has more CHO levels, which can provide rich carbohydrates for ruminants [34]. CA is a rapidly fermenting water-soluble consisting mainly of sugars but also containing organic acids and short oligosaccharides [34]. In this study, the CA levels of Zhaozhou industrial hemp ethanol extraction byproduct and alfalfa hay were similar, significantly lower than Daxing’ anling industrial hemp ethanol extraction byproduct, and both could be rapidly degraded. The higher level of CB1 in Daxing’ anling industrial hemp ethanol extraction byproduct is related to the higher starch content with a slower degradation rate than CA [34]. Industrial hemp stalk and alfalfa hay have the same CB2 portion, meaning their available NDF ferments more slowly but is still potentially available [34]. The high CC fraction of industrial hemp byproducts can still limit their application in practical production due to the indigestible fraction in the rumen.

### 4.3. In Situ Ruminal Degradation

The rumen degradation rate of DM can affect the intake of dry matter in livestock, which is an important index for evaluating the nutritional value of feed [36]. It depends on the content of cellulose and the lignification degree of feed raw materials and can reflect the difficulty of digestion of feed [36]. Industrial hemp oil filter residue has a higher effective DM and CP degradation rate related to its higher rapid degradation part. Excessive supplementation of lipids may negatively affect rumen fermentation, affecting fiber digestion and dry matter intake, so attention should be paid to the amount of industrial hemp oil filter residue when applied [37]. Daxing’ anling industrial hemp ethanol extraction byproduct has lower *ED* values of CP rumen degradation, which may be related to its high content of NDICP and ADICP. In feed nutrients, NDICP cannot be degraded in the rumen but is digested and absorbed in the small intestine, providing rumen bypass protein for dairy cows [38]. Industrial hemp seed meal has similar *a* + *b* values of CP rumen degradation to soybean meal, but less *ED* makes it more RUP. This indicates that industrial hemp seed meal is an excellent natural source of non-degradable rumen proteins, consistent with the findings of Mustafa et al. [8]. Similarly, the *in situ* CP rumen degradation rates of industrial hemp ethanol extraction byproducts and industrial hemp stalk is lower compared with alfalfa hay, and more proteins can reach the hind digestive tract through the rumen for digestion and absorption. However, specific nutrient utilization efficiency will be further discussed in combination with small intestinal digestibility later. The effective degradability of NDF of Daxing’ anling industrial hemp ethanol extraction byproduct is higher than that of alfalfa hay, corresponding to its high CA component and *c* value of NDF rumen degradation. Therefore, Daxing’ anling industrial hemp ethanol extraction byproduct can provide abundant carbohydrates for ruminants and maintain normal rumen fermentation.

### 4.4. Intestinal Digestion

For ruminants, excessive protein is degraded in the rumen, leading to insufficient protein entering the small intestine, which cannot meet the nutritional requirements of ruminants having fast growth and high protein demand [39]. Therefore, high-quality feed should make most proteins available to reach the small intestine for digestion and utilization [39]. Soybean meal still has a high degradation rate in the degraded part of the small intestine, which is worthy of being widely used as conventional feed [40]. Compared with soybean meal, industrial hemp seed meal has the same IDP value and less TTDP value, so it can be considered as the concentrate feed component of dairy cows in practical production. In addition, Mustafa et al. [8] showed that the digestibility of industrial hemp seed meal was equal to that of canola meal and could be used as a substitute for canola meal without adverse effects on nutrient utilization in sheep. The high TTDP value and CP rumen degradation rate of industrial hemp oil filter residue make it possible to provide easily degradable protein for lactating cows. The TTDP value of Zhaozhou industrial hemp ethanol extraction byproduct is second only to alfalfa hay and has more CP content, so it can be considered to provide rich protein nutrition for ruminants.

### 4.5. Energy Value Estimation

According to NASEM (2021) [27], the feed was separated into more fractions: NDF, starch, FA, CP, ash, and ROM. ROM is estimated to be predominantly sugars, organic acids (mostly lactic and acetic), glycerol, and soluble fiber. The DE calculations for dairy cattle are based on the NASEM [27] requirement for cows consuming DM at 3.5% of body weight and fed a diet with 26% starch and 30% NDF. The energy values of alfalfa hay and soybean meal in this experiment were smaller than those in NASEM [27,28], which is related to the different chemical compositions of their feed. The results of this experiment show that industrial hemp oil filter residue can provide substantial energy to cattle while Daxing’ anling industrial hemp ethanol extraction byproduct has the least energy value compared with alfalfa hay. These energy values serve as a useful reference for cattle feed formulation.

## 5. Conclusions

Different industrial hemp byproducts’ nutritional values and degradation characteristics were significantly different. Based on the analytical data, we concluded that industrial hemp ethanol extraction byproduct had higher CP and ruminal potential NDF degradation, industrial hemp stalk had higher CHO, industrial hemp seed meal can provide rumen protection proteins, and industrial hemp oil filter residue can provide more rapidly degrading proteins. Industrial hemp byproducts can be considered for inclusion in dietary formulations as unconventional feed sources for dairy cattle, but the purpose of use needs to be properly considered.

## Figures and Tables

**Table 1:** Chemical composition of industrial hemp byproducts and conventional feeds.

Item	ZIHEEB	DIHEEB	IHS	AH	SEM	*p*	IHSM	IHOFR	SBM	SEM	*p*
DM (g/kg)	859 ^d^	875 ^c^	937 ^a^	927 ^b^	0.82	<0.01	917 ^a^	912 ^b^	888 ^c^	0.68	<0.01
Ash (g/kg DM)	212 ^a^	189 ^b^	84.8 ^d^	89.7 ^c^	1.09	<0.01	83.3 ^b^	128 ^a^	73.1 ^c^	0.54	<0.01
EE (g/kg DM)	52.5 ^a^	15.1 ^c^	27.3 ^b^	31.4 ^b^	1.62	<0.01	79.2 ^b^	216 ^a^	22.9 ^c^	0.95	<0.01
CP (g/kg DM)	208 ^a^	172 ^b^	91.2 ^d^	162 ^c^	1.89	<0.01	296 ^b^	442 ^a^	445 ^a^	3.38	<0.01
SCP (g/kg CP)	132 ^c^	60.4 ^d^	265 ^b^	426 ^a^	10.2	<0.01	96.0 ^b^	192 ^a^	189 ^a^	7.24	<0.01
NPN (g/kg SCP)	791 ^b^	339 ^c^	945 ^a^	812 ^b^	28.0	<0.01	378 ^a^	405 ^a^	70.0 ^b^	54.2	<0.01
NDICP (g/kg CP)	503 ^b^	690 ^a^	413 ^c^	246 ^d^	14.4	<0.01	329 ^a^	263 ^b^	285 ^ab^	15.0	0.03
ADICP (g/kg CP)	191 ^b^	376 ^a^	200 ^b^	93.0 ^c^	9.93	<0.01	103 ^a^	23.0 ^b^	12.9 ^c^	1.62	<0.01
NDF (g/kg DM)	471 ^c^	460 ^c^	629 ^a^	532 ^b^	8.89	<0.01	536 ^a^	201 ^c^	292 ^b^	7.52	<0.01
ADF (g/kg DM)	303 ^c^	310 ^c^	470 ^a^	376 ^b^	6.43	<0.01	356 ^a^	89.9 ^b^	89.0 ^b^	2.53	<0.01
ADL (g/kg DM)	118 ^a^	125 ^a^	116 ^a^	103 ^b^	3.65	<0.01	146 ^a^	29.1 ^b^	16.5 ^c^	2.66	<0.01
Starch (g/kg DM)	3.12 ^d^	48.1 ^a^	18.8 ^b^	16.8 ^c^	0.46	<0.01	1.34 ^c^	2.72 ^b^	22.9 ^a^	0.19	<0.01
NFC (g/kg DM)	55.4 ^b^	163 ^a^	168 ^a^	185 ^a^	8.16	<0.01	5.16 ^b^	13.2 ^b^	167 ^a^	7.38	<0.01
Ca (g/kg DM)	35.5 ^b^	44.6 ^a^	13.3 ^d^	16.3 ^c^	0.89	<0.01	3.64 ^a^	2.65 ^b^	3.87 ^a^	0.14	<0.01
P (g/kg DM)	9.69 ^a^	7.53 ^b^	5.32 ^c^	2.92 ^d^	0.08	<0.01	11.7 ^b^	20.8 ^a^	6.25 ^c^	0.24	<0.01
THC (g/kg DM)	<LOQ	<LOQ	<LOQ	-			-	-	-		

ZIHEEB, Zhaozhou industrial hemp ethanol extraction byproduct; DIHEEB, Daxing’ anling industrial hemp ethanol extraction byproduct; IHS, industrial hemp stalk; AH, alfalfa hay; IHSM, industrial hemp seed meal; IHOFR, industrial hemp oil filter residue; SBM, soybean meal. DM, dry matter; EE, ether extract; CP, crude protein; SCP, soluble crude protein; NPN, non-protein nitrogen; NDICP, neutral detergent insoluble crude protein; ADICP, acid detergent insoluble crude protein; NDF, neutral detergent fiber; ADF, acid detergent fiber; ADL, acid detergent lignin; NFC, non-fiber carbohydrate; THC, tetrahydrocannabinol; LOQ, lower limit of quantification. ^a–d^ Values in the same line with different capital letters superscripts mean samples have a significant difference, the same as below. SEM, the standard error of the mean, is the same as below.

**Table 2:** Crude protein and carbohydrate fractions of the industrial hemp byproducts and conventional feeds according to the Cornell Net Carbohydrate and Protein System (CNCPS).

Item	ZIHEEB	DIHEEB	IHS	AH	SEM	*p*	IHSM	IHOFR	SBM	SEM	*p*
Protein fractions (g/kg CP)											
PA	104 ^c^	19.4 ^d^	250 ^b^	346 ^a^	10.4	<0.01	35.7 ^b^	76.3 ^a^	13.2 ^c^	5.29	<0.01
PB1	27.7 ^bc^	41.0 ^b^	14.4 ^c^	80.2 ^a^	6.01	<0.01	60.4 ^c^	116 ^b^	175 ^a^	9.80	<0.01
PB2	365 ^a^	250 ^b^	323 ^a^	328 ^a^	17.1	<0.01	575 ^a^	545 ^ab^	526 ^b^	14.4	<0.01
PB3	312 ^a^	314 ^a^	212 ^b^	153 ^c^	16.3	<0.01	226	240	272	14.1	0.09
PC	191 ^b^	376 ^a^	200 ^b^	92.6 ^c^	9.93	<0.01	103 ^a^	23.0 ^b^	12.9 ^c^	1.62	<0.01
CHO (g/kg DM)	527 ^d^	623 ^c^	797 ^a^	717 ^b^	2.55	<0.01	541 ^a^	214 ^c^	459 ^b^	3.75	<0.01
NSC (g/kg CHO)	305 ^b^	452 ^a^	258 ^c^	314 ^b^	12.5	<0.01	189 ^c^	603 ^b^	641 ^a^	9.50	<0.01
Carbohydrate fractions (g/kg CHO)											
CA	299 ^b^	375 ^a^	234 ^c^	290 ^b^	12.3	<0.01	187 ^b^	590 ^a^	591 ^a^	9.64	<0.01
CB1	5.92 ^c^	77.1 ^a^	23.6 ^b^	23.4 ^b^	0.64	<0.01	2.46 ^c^	12.7 ^b^	50.0 ^a^	0.34	<0.01
CB2	156 ^b^	66.9 ^c^	392 ^a^	343 ^a^	18.3	<0.01	162 ^b^	71.0 ^c^	273 ^a^	19.1	<0.01
CC	540 ^a^	481 ^b^	350 ^c^	344 ^c^	15.9	<0.01	649 ^a^	326 ^b^	86.2 ^c^	12.1	<0.01

ZIHEEB, Zhaozhou industrial hemp ethanol extraction byproduct; DIHEEB, Daxing’ anling industrial hemp ethanol extraction byproduct; IHS, industrial hemp stalk; AH, alfalfa hay; IHSM, industrial hemp seed meal; IHOFR, industrial hemp oil filter residue; SBM, soybean meal. PA, non-protein nitrogen; PB1, rapidly degraded protein; PB2, intermediately degraded protein; PB3, slowly degraded protein; PC, unavailable protein; CHO, carbohydrate; NSC, non-structural carbohydrate; CA, sugars and soluble fraction; CB1, starch and pectin; CB2, fraction available cell wall; CC, unavailable cell wall. ^a–d^ Values in the same line with different capital letter superscripts mean samples have significant differences. SEM, standard error of the mean.

**Table 3:** *In situ* ruminal degradation kinetics in industrial hemp byproducts and conventional feeds.

Item	ZIHEEB	DIHEEB	IHS	AH	SEM	*p*	IHSM	IHOFR	SBM	SEM	*p*
*In situ* dry matter (DM) rumen degradation kinetics											
*a* (g/kg)	237 ^a^	213 ^b^	163 ^c^	248 ^a^	5.16	<0.01	184 ^c^	559 ^a^	268 ^b^	7.45	<0.01
*b* (g/kg)	596 ^c^	746 ^a^	652 ^b^	483 ^d^	10.3	<0.01	643 ^b^	260 ^c^	700 ^a^	11.2	<0.01
*a* + *b* (g/kg)	834 ^b^	959 ^a^	815 ^b^	731 ^c^	7.20	<0.01	826 ^b^	820 ^b^	968 ^a^	12.2	<0.01
*c* (g/kg h^−1^)	23.9 ^b^	22.8 ^b^	15.2 ^b^	63.9 ^a^	2.83	<0.01	12.9 ^c^	43.3 ^a^	37.0 ^b^	1.68	<0.01
*ED* (g/kg)	444 ^c^	463 ^b^	327 ^d^	530 ^a^	4.80	<0.01	297 ^c^	668 ^a^	534 ^b^	2.44	<0.01
*In situ* crude protein (CP) rumen degradation kinetics											
*a* (g/kg)	247 ^b^	77.8 ^c^	398 ^a^	227 ^b^	15.7	<0.01	209 ^b^	628 ^a^	230 ^b^	6.83	<0.01
*b* (g/kg)	670 ^b^	816 ^a^	511 ^c^	635 ^b^	26.8	<0.01	725 ^a^	295 ^b^	728 ^a^	15.9	<0.01
*a* + *b* (g/kg)	917	894	909	862	16.6	0.18	934	923	958	16.2	0.36
*c* (g/kg h^−1^)	31.1 ^bc^	35.0 ^b^	18.4 ^c^	90.4 ^a^	4.52	<0.01	35.9 ^c^	71.1 ^a^	43.4 ^b^	2.00	<0.01
*ED* (g/kg)	520 ^c^	435 ^d^	547 ^b^	650 ^a^	6.36	<0.01	480 ^c^	788 ^a^	536 ^b^	4.22	<0.01
RUP (g/kg)	480 ^b^	565 ^a^	453 ^c^	350 ^d^	6.36	<0.01	520 ^a^	212 ^c^	464 ^b^	4.22	<0.01
*In situ* neutral detergent fiber (NDF) rumen degradation kinetics											
*a* (g/kg)	124 ^a^	72.7 ^b^	13.9 ^c^	79.7 ^b^	6.49	<0.01	27.0 ^c^	171 ^b^	238 ^a^	4.85	<0.01
*b* (g/kg)	726 ^ab^	767 ^a^	578 ^c^	677 ^b^	17.1	<0.01	191 ^c^	460 ^b^	621 ^a^	14.3	<0.01
*a* + *b* (g/kg)	850 ^a^	840 ^a^	591 ^c^	757 ^b^	11.0	<0.01	218 ^c^	630 ^b^	859 ^a^	18.0	<0.01
*c* (g/kg h^−1^)	15.5 ^c^	32.4 ^a^	15.9 ^c^	24.5 ^b^	0.57	<0.01	21.2 ^c^	72.6 ^a^	52.5 ^b^	3.54	<0.01
*ED* (g/kg)	311 ^b^	394 ^a^	165 ^c^	318 ^b^	4.32	<0.01	73.7 ^c^	422 ^b^	527 ^a^	1.08	<0.01

ZIHEEB, Zhaozhou industrial hemp ethanol extraction byproduct; DIHEEB, Daxing’ anling industrial hemp ethanol extraction byproduct; IHS, industrial hemp stalk; AH, alfalfa hay; IHSM, industrial hemp seed meal; IHOFR, industrial hemp oil filter residue; SBM, soybean meal. *a*, rapidly degradable fraction in rumen degradation; *b*, slowly degradable fraction in rumen degradation; *a + b*, potentially degradable fraction in rumen degradation; *c*, the degradation rate of the slowly degradable fraction; *ED*, effective degradability of the incubated samples; RUP, rumen undegradable protein. ^a–d^ Values in the same line with different capital letter superscripts mean samples have significant differences. SEM, standard error of the mean.

**Table 4:** The intestinal degradation ratio of industrial hemp byproducts and conventional feeds.

Item	ZIHEEB	DIHEEB	IHS	AH	SEM	*p*	IHSM	IHOFR	SBM	SEM	*p*
IDDM (g/kg RUDM)	196 ^b^	165 ^c^	80.9 ^d^	226 ^a^	6.30	<0.01	285 ^b^	310 ^b^	646 ^a^	14.8	<0.01
IDRUP (g/kg RUP)	209 ^b^	139 ^c^	177 ^bc^	599 ^a^	11.8	<0.01	751 ^c^	837 ^b^	878 ^a^	7.25	<0.01
IDP (g/kg CP)	100 ^b^	78.8 ^b^	80.4 ^b^	209 ^a^	6.48	<0.01	391 ^a^	178 ^b^	408 ^a^	5.53	<0.01
TTDP (g/kg CP)	620 ^b^	514 ^c^	628 ^b^	860 ^a^	4.19	<0.01	871 ^c^	965 ^a^	943 ^b^	3.63	<0.01

ZIHEEB, Zhaozhou industrial hemp ethanol extraction byproduct; DIHEEB, Daxing’ anling industrial hemp ethanol extraction byproduct; IHS, industrial hemp stalk; AH, alfalfa hay; IHSM, industrial hemp seed meal; IHOFR, industrial hemp oil filter residue; SBM, soybean meal. IDDM, intestinal digested dry matter; RUDM, rumen undegradable dry matter; IDRUP, intestinal digested rumen undegradable protein; RUP, rumen undegradable protein; IDP, intestinal digested protein; CP, crude protein; TTDP, total tract digested protein. ^a–d^ Values in the same line with different capital letter superscripts mean samples have significant differences. SEM, standard error of the mean.

**Table 5:** The estimated digestible energy values of industrial hemp byproducts and conventional feeds.

Item	ZIHEEB	DIHEEB	IHS	AH	SEM	*p*	IHSM	IHOFR	SBM	SEM	*p*
Feed fractions (NASEM, 2021)											
FA (g/kg of DM)	29.8 ^a^	8.55 ^c^	15.5 ^b^	17.8 ^b^	0.92	<0.01	69.2 ^b^	206 ^a^	12.9 ^c^	0.95	<0.01
ROM (g/kg of DM)	90.5 ^d^	124 ^c^	176 ^b^	219 ^a^	8.38	<0.01	24.6 ^c^	53.7 ^b^	158 ^a^	7.93	<0.01
GE (MJ/kg)	15.5 ^b^	15.4 ^c^	16.6 ^a^	16.7 ^a^	0.04	<0.01	19.4 ^b^	22.4 ^a^	19.1 ^c^	0.04	<0.01
GE_b_ (MJ/kg)	16.6 ^c^	16.3 ^d^	17.8 ^b^	18.4 ^a^	0.02	<0.01	21.0 ^b^	23.7 ^a^	19.5 ^c^	0.02	<0.01
True digestibility coefficients and digestible energy for dairy cattle (NASEM, 2021)											
dNDF (g/kg of NDF)	338 ^b^	318 ^b^	413 ^a^	403 ^a^	8.49	<0.01	316 ^c^	465 ^b^	603 ^a^	7.73	<0.01
dCP (g/kg of CP)	620 ^b^	514 ^c^	628 ^b^	860 ^a^	4.19	<0.01	871 ^c^	965 ^a^	943 ^b^	3.63	<0.01
DE (MJ/kg of DM)	6.46 ^c^	6.28 ^c^	7.71 ^b^	8.91 ^a^	0.10	<0.01	9.94 ^c^	16.5 ^a^	14.9 ^b^	0.08	<0.01
Predicted energy value for beef cattle (NASEM, 2016)											
TDN (g/kg of DM)	447 ^c^	407 ^d^	478 ^b^	533 ^a^	6.86	<0.01	584 ^c^	979 ^a^	777 ^b^	3.53	<0.01
DE (MJ/kg of DM)	8.25 ^c^	7.51 ^d^	8.81 ^b^	9.83 ^a^	0.13	<0.01	10.8 ^c^	18.1 ^a^	14.3 ^b^	0.07	<0.01
ME (MJ/kg of DM)	6.77 ^c^	6.16 ^d^	7.23 ^b^	8.06 ^a^	0.10	<0.01	8.84 ^c^	14.8 ^a^	11.8 ^b^	0.05	<0.01
NE_m_ (MJ/kg of DM)	3.26 ^c^	2.64 ^d^	3.72 ^b^	4.53 ^a^	0.10	<0.01	5.26 ^c^	10.3 ^a^	7.84 ^b^	0.05	<0.01
NE_g_ (MJ/kg of DM)	1.01 ^c^	0.43 ^d^	1.45 ^b^	2.21 ^a^	0.10	<0.01	2.88 ^c^	7.27 ^a^	5.18 ^b^	0.04	<0.01

ZIHEEB, Zhaozhou industrial hemp ethanol extraction byproduct; DIHEEB, Daxing’ anling industrial hemp ethanol extraction byproduct; IHS, industrial hemp stalk; AH, alfalfa hay; IHSM, industrial hemp seed meal; IHOFR, industrial hemp oil filter residue; SBM, soybean meal. FA, fatty acid; ROM, residual organic matter; GE, gross energy; GE_b_, gross energy (bomb calorimeter); dNDF, the digested proportion of NDF; dCP, the digested proportion of CP; DE, digestible energy; TDN, total digestible nutrient; ME, metabolic energy; NE_m_, net energy for maintenance; NE_g_, net energy for gain. ^a–d^ Values in the same line with different capital letter superscripts mean samples have significant differences. SEM, standard error of the mean.

## Data Availability

The data presented in this study are available on request from the corresponding author.

## References

[1] Capanoglu E., Nemli E., Tomas-Barberan F. (2022). Novel Approaches in the Valorization of Agricultural Wastes and Their Applications. J. Agric. Food Chem..

[2] Bradford B.J., Mullins C.R. (2012). Invited review: Strategies for promoting productivity and health of dairy cattle by feeding nonforage fiber sources. J. Dairy Sci..

[3] Russo E.B. (2007). History of cannabis and its preparations in saga, science, and sobriquet. Chem. Biodivers..

[4] Semwogerere F., Katiyatiya C.L.F., Chikwanha O.C., Marufu M.C., Mapiye C. (2020). Bioavailability and bioefficacy of hemp byproducts in ruminant meat production and preservation: A review. Front. Vet. Sci..

[5] Capanoglu E., Tomás-Barberán F.A. (2022). Introduction to Novel Approaches in the Valorization of Agricultural Wastes and Their Applications. J. Agric. Food Chem..

[6] Schluttenhofer C., Yuan L. (2017). Challenges towards revitalizing hemp: A multifaceted crop. Trends Plant Sci..

[7] Karlsson L., Finell M., Martinsson K. (2010). Effects of increasing amounts of hempseed cake in the diet of dairy cows on the production and composition of milk. Animal.

[8] Mustafa A.F., McKinnon J.J., Christensen D.A. (1999). The nutritive value of hemp meal for ruminants. Can. J. Anim. Sci..

[9] Mierliță D. (2018). Effects of diets containing hemp seeds or hemp cake on fatty acid composition and oxidative stability of sheep milk. S. Afr. J. Anim. Sci..

[10] Kleinhenz M.D., Magnin G., Ensley S.M., Griffin J.J., Goeser J., Lynch E., Coetzee J.F. (2020). Nutrient concentrations, digestibility, and cannabinoid concentrations of industrial hemp plant components. Appl. Anim. Sci..

[11] Parker N.B., Bionaz M., Ford H.R., Irawan A., Trevisi E., Ates S. (2022). Assessment of spent hemp biomass as a potential ingredient in ruminant diet: Nutritional quality and effect on performance, meat and carcass quality, and hematological parameters in finishing lambs. J. Anim. Sci..

[12] Altman A.W., Vanzant E.S., McLeod K.R., Harmon D.L. (2022). In Vitro Measurements of True Digestibility and Products of Digestion Using Multiple Cultivars of Non-Extracted and CBD-Extracted Industrial Hemp Biomass (*Cannabis sativa*). Front. Anim. Sci..

[13] Fox D.G., Barry M.C., Pitt R.E., Roseler D.K., Stone W.C. (1995). Application of the Cornell net carbohydrate and protein model for cattle consuming forages. J. Anim. Sci..

[14] Harper K.J., McNeill D.M. (2015). The role iNDF in the regulation of feed intake and the importance of its assessment in subtropical ruminant systems (the role of iNDF in the regulation of forage intake). Agriculture.

[15] Wang E.D., Wang J.D., Lv J.Y., Sun X.G., Kong F.L., Wang S., Wang Y.J., Yang H.J., Cao Z.J., Li S.L. (2021). Comparison of Ruminal Degradability, Indigestible Neutral Detergent Fiber, and Total-Tract Digestibility of Three Main Crop Straws with Alfalfa Hay and Corn Silage. Animals.

[16] Tian Y.J., Zhang X.W., Li S.L., Liu K., Guo P. (2019). Effect of Harvest Time and Microbial Anaerobic Fermentation at Ruminal Degradability, In Vitro Digestibility to Milk Production and Milk Quality for Whole Plant Zhang Hybrid Millet in Dairy Cows. Animals.

[17] AOAC (2000). Official Methods of Analysts.

[18] Licitra G., Hernandez T., Van Soest P.J. (1996). Standardization of procedures for nitrogen fractionation of ruminant feeds. Anim. Feed Sci. Technol..

[19] Van Soest P.J., Robertson J.B., Lewis B.A. (1991). Methods for dietary fiber, neutral detergent fiber, and nonstarch polysaccharides in relation to animal nutrition. J. Dairy Sci..

[20] Hädener M., König S., Weinmann W. (2019). Quantitative determination of CBD and THC and their acid precursors in confiscated cannabis samples by HPLC-DAD. Forensic Sci. Int..

[21] Sniffen C.J., O’Connor J.D., Van Soest P.J., Fox D.G., Russell J.B. (1992). A net carbohydrate and protein system for evaluating cattle diets: II. Carbohydrate and protein availability. J. Anim. Sci..

[22] Li Y., Wu Q.H., Lv J.Y., Jia X.M., Gao J.X., Zhang Y.G., Wang L. (2022). Associations of Protein Molecular Structures with Their Nutrient Supply and Biodegradation Characteristics in Different Byproducts of Seed-Used Pumpkin. Animals.

[23] Mjoun K., Kalscheur K.F., Hippen A.R., Schingoethe D.J. (2010). Ruminal degradability and intestinal digestibility of protein and amino acids in soybean and corn distillers grains products. J. Dairy Sci..

[24] Ørskov E.-R., McDonald I. (1979). The estimation of protein degradability in the rumen from incubation measurements weighted according to rate of passage. J. Agric. Sci..

[25] Tamminga S., Van Straalen W.M., Subnel A.P.J., Meijer R.G.M., Steg A., Wever C.J.G., Blok M.C. (1994). The Dutch protein evaluation system: The DVE/OEB-system. Livest. Prod. Sci..

[26] Calsamiglia S., Stern M.D. (1995). A three-step in vitro procedure for estimating intestinal digestion of protein in ruminants. J. Anim. Sci..

[27] National Academies of Sciences, Engineering, and Medicine (2021). Nutrient Requirements of Dairy Cattle.

[28] National Academies of Sciences, Engineering, and Medicine (2016). Nutrient Requirements of Beef Cattle.

[29] Bernstein N., Gorelick J., Zerahia R., Koch S. (2019). Impact of N, P, K, and humic acid supplementation on the chemical profile of medical cannabis (*Cannabis sativa* L). Front. Plant Sci..

[30] Bailoni L., Bacchin E., Trocino A., Arango S. (2021). Hemp (*Cannabis sativa* L.) seed and co-products inclusion in diets for dairy ruminants: A review. Animals.

[31] Serrapica F., Masucci F., Raffrenato E., Sannino M., Vastolo A., Barone C.M.A., Di Francia A. (2019). High fiber cakes from Mediterranean multipurpose oilseeds as protein sources for ruminants. Animals.

[32] Yang P., Ni J.J., Zhao J.B., Zhang G., Huang C.F. (2020). Regression equations of energy values of corn, soybean meal, and wheat bran developed by chemical composition for growing pigs. Animals.

[33] EFSA Panel on Additives and Products or Substances used in Animal Feed (FEEDAP) (2011). Scientific Opinion on the safety of hemp (*Cannabis* genus) for use as animal feed. EFSA J..

[34] Fox D.G., Tedeschi L.O., Tylutki T.P., Russell J.B., Van Amburgh M.E., Chase L.E., Pell A.N., Overton T.R. (2004). The Cornell Net Carbohydrate and Protein System model for evaluating herd nutrition and nutrient excretion. Anim. Feed Sci. Technol..

[35] Higgs R., Chase L.E., Ross D.A., Van Amburgh M.E. (2015). Updating the Cornell Net Carbohydrate and Protein System feed library and analyzing model sensitivity to feed inputs. J. Dairy Sci..

[36] Li Q., Xue B., Zhao Y.M., Wu T.Q., Liu H.C., Yi X., Sun C.C., Wang Z.S., Zou H.W., Yan T.H. (2018). In situ degradation kinetics of 6 roughages and the intestinal digestibility of the rumen undegradable protein. J. Anim. Sci..

[37] Fiorentini G., Carvalho I.P.C., Messana J.D., Canesin R.C., Castagnino P.S., Lage J.F., Arcuri P.B. (2015). Effect of lipid sources with different fatty acid profiles on intake, nutrient digestion and ruminal fermentation of feedlot Nellore steers. Asian-Australas. J. Anim. Sci..

[38] Yu P., Nuez-Ortín W.G. (2010). Relationship of protein molecular structure to metabolizable proteins in different types of dried distillers grains with solubles: A novel approach. Br. J. Nutr..

[39] Chalupa W., Sniffen C.J. (1991). Protein and amino acid nutrition of lactating dairy cattle. The Veterinary Clinics of North America. Food Anim. Pract..

[40] Paula E.M., Broderick G.A., Faciola A.P. (2020). Effects of replacing soybean meal with canola meal for lactating dairy cows fed 3 different ratios of alfalfa to corn silage. J. Dairy Sci..

